# Recognition of everyday activities using experiment data from wearable sensors: a deep learning-based framework

**DOI:** 10.1038/s41598-026-63774-8

**Published:** 2026-07-24

**Authors:** FrameworkWilliam Son Galanza, Steven M. Schmidt, Sofi Fristedt, Nebojsa Malesevic

**Affiliations:** 1 Department of Health Sciences, Faculty of Medicine, Lund University, Lund, Sweden; 2 School of Health and Welfare, Jönköping University, Jönköping, Sweden; 3 Department of Biomedical Engineering, Faculty of Engineering, Lund University, Lund, Sweden

**Keywords:** Human activity recognition, Deep learning algorithms, Health monitoring systems, Older adult participation and mobility, Experiences of older adults, User experience, Engineering, Health care, Mathematics and computing

## Abstract

Tracking everyday activities is vital for detecting changes in older adults’ health, allowing timely support to promote well-being. Wearable sensors and deep learning provide continuous monitoring, making them a supportive tool in detecting such changes. However, a more refined method is needed to recognise precise activities with a minimal set of sensors. This study aimed to develop a method to recognise everyday activities among older adults by utilising wearable sensors and a deep learning model. This is a small-scale home lab experiment to develop a method to recognise 14 everyday activities. We compared five models that recognised everyday activities with different sensor signal counts and accuracy. Our results showed that sensor placement is important. Based on the results, we proposed a two-sensor method (pelvis and right hand) to collect and correctly recognise everyday activities among older adults. This model, which utilises two sensors, classified 12 activities with an accuracy of 89.3%. Another model recognised all 14 activities with a lower accuracy of 88.2% using five sensors. We also explored a one-sensor approach, which showed low recognition performance and struggled to distinguish activity variability. The two-sensor-based system will allow for large-scale data collection on everyday activities of older adults.

**Supplementary Information:**

The online version contains supplementary material available at 10.1038/s41598-026-63774-8.

## Introduction

Wearable sensors are among the primary technologies used to collect data on human activities across various settings, including healthcare, fitness, and sports^1,2^. Their ability to provide real-time, continuous monitoring makes sensors a supportive tool for older adults´ independence and security, with the potential to identify health issues. Engagement in activities is important for maintaining positive well-being^3,4^. Studies have shown that consistent engagement in activities promotes better physical health, cognitive function, and emotional well-being^5,6^. However, as people age, the incidence of physical performance limitations will increase as well^7^. These physical limitations predispose older adults to fewer opportunities for activities^7,8^. Sedentary behaviour among older adults is a significant health concern linked to increased risks of chronic diseases, reduced mobility, and lower quality of life^9–11^. Monitoring everyday activities, such as walking, sitting, cooking, and taking medication, over time can support the identification of age-related changes, including shift towards sedentary routines or altered gait pattern, which may indicate the onset of conditions like depression or chronic diseases^12^. Assessing everyday activities through home monitoring in older adults can significantly contribute to the early prediction of disease progression, leading to more effective prevention or treatment^13,14^.

Older adults typically spend substantial time in the home environment, where gradual changes in health status or functional capacity may go unnoticed until they lead to adverse outcomes. The amount of time spent within the home generally increases as people age, boosting the importance of the home environment to support a range of activities and monitoring^15^. Most older adults prefer to live safely and autonomously in their familiar home environment as long as possible^15^. The World Health Organisation estimates that 1.8 billion adults face health risks due to insufficient activity, including cardiovascular diseases and dementia^9^. As age demographic distribution continues to surge among the older age group^9^, the need for sensor-based in-home monitoring is becoming more important to support independent living, manage age-related health conditions, and alleviate pressure on healthcare systems^16^.

Several conventional monitoring methods exist to evaluate older adults’ functioning in everyday activities at home and in clinical settings. These include self-report and clinical assessments based on observation by qualified healthcare professionals. However, self-reports are subjective, potentially influenced by recall bias and may not give a complete picture of the person’s functional performance^14,16^. Conventional assessment is usually initiated at a health facility when problems such as confusion, falls, immobility, or incontinence are prominent. Thus, the condition may have already progressed, and the intervention is offered late in the process. Sensor-based systems, on the other hand, offer objective, continuous, and comprehensive data, enabling the early identification of individual changes in functioning and health problems, which could support individual rehabilitation or care plans^17–19^.

Recent advances highlight the effectiveness of sensor technologies in monitoring and recognising activities^20–23^. However, a more refined method is needed to precisely distinguish between different everyday activities and the types of sensors included within current monitoring methods^14,24^. The reliability of sensor data in real-world environments remains a challenge. Factors like sensor placement, environmental interference, and noise can degrade data quality, affecting activity recognition accuracy. Algorithm methods that mitigate these issues are essential to ensure reliable performance in recognising diverse everyday activities and settings^24^.

Machine learning (ML) has been transformative in analysing high-dimensional sensor data for activity monitoring. Early approaches relied on traditional ML techniques such as k-nearest neighbours, decision trees, and support vector machines, which used manually engineered features derived from sensor data^25–27^. While these methods showed initial promise, they often struggled with generalisation across diverse activities and required substantial domain expertise for feature engineering. Deep learning (DL) has addressed these limitations by enabling models to learn feature representations directly from signal features^26,27^. Hence, DL methods can be applied to activity monitoring using sensors to allow precise activity recognition, potentially.

Despite its potential, activity monitoring for older adults is still underdeveloped. Real-world applications face challenges such as technology acceptance, obtrusiveness, lighting variations, occlusions, and background noise associated with vision- and audio-based systems (e.g., cameras and microphones), which can undermine reliability^26,27^. Another issue is the variability in how individuals perform the same activity^28^. The range of everyday activities may display subtle differences influenced by current health status and health history, as well as older adults’ preferences and capability to perform each activity^27,29^. Also, factors such as age, mobility limitations, health conditions, and household settings complicate the development of models for activity recognition^23,27^.

The feasibility and effectiveness of sensor technological innovation to gather data in the homes of older adults have been explored^23^. However, further studies in different settings and the utilisation of other data, such as everyday activity, are still needed^30^. Furthermore, datasets used to train activity recognition models involving older adults are scarce. There is a need for methods that can monitor everyday activities more directly and richly with the older adults as participants^23^. However, user acceptance can pose another challenge to adoption. Individuals may resist using wearable devices or environmental sensors due to discomfort, unfamiliarity, or privacy concerns^31^. Ensuring these technologies are user-friendly, non-intrusive, and respectful of privacy is essential for widespread adoption. Moreover, robust data protection mechanisms, such as encryption and secure storage, are critical to alleviating concerns about privacy and security, mainly when monitoring occurs in private living spaces^27,30^. Nevertheless, using sensor technologies in home environments is a complex process that requires a contextual understanding of older adults’ experience with such technologies in the home setting. This study aimed to develop a method to accurately recognise everyday activities among older adults by utilising wearable sensors. We specifically aim to (1) develop a DL algorithm that can correctly recognise different everyday activities, (2) identify the minimal number of sensors to collect data on everyday activities while maintaining a robust DL model, and (3) explore participants’ experience of the use of sensors.

## Methods

This is a small-scale experiment to develop a method to recognise everyday activities using wearable sensors. We also explored participants’ experiences with sensors through a short questionnaire, including design, privacy, comfort, usability, and willingness to continue using them.

We affirm adherence to the principles of ethical and professional conduct within the study and confirm that all methods were carried out in accordance with relevant guidelines and regulations. The Swedish Ethical Review Authority approved the study (No: 2024-02004-01).

### Participants and recruitment

Participants were recruited by sending invitations to existing mailing lists (including people who had expressed interest in participating in research and other activities). A leaflet was distributed digitally through contacts and posted in public areas, such as universities and library receptions. People who were interested made contact via email or telephone, received an information letter via email, and were subsequently contacted by the researchers by telephone or mail. The inclusion criteria were being age 65 years and older, feeling physically fit to participate in the study, able to travel to the study setting, willing to wear sensors, and able to perform 14 everyday activities. Participants who met these criteria and volunteered to participate were scheduled on a date and time of their preference.

Ten older adults gave informed consent to participate, including consent for audio and video recordings. Before each session, all participants were encouraged to raise concerns (none were expressed) and ask questions about the study. The participants displayed moderate diversity in terms of gender, civil status, and income (Table 1). Given that this was an exploratory, small-scale experiment focused on method development and sensor configuration rather than on population-level generalisation, a sample of ten older adults was deemed acceptable.

**Table 1:** Participant characteristics (*N* = 10).

Demographic variable	Total
*Age, years*	
70–79	8
80–89	2
*Gender*	
Men	3
Women	7
*Education*	
High school and below	2
University	8
*Civil status*	
Single	5
Partnered	5
*Income/ Month (Swedish krona)*	
< 20,000–29,000	7
30,000–39,000	3
*Health status*	
Good	5
Excellent	5
*Smart home technology experience*	
Yes	3
No	7

### Study setting

The data were collected in a controlled environment to ensure consistency and minimise variability, i.e., the Movement and Reality Lab (MoRe-Lab) at the Faculty of Medicine, Lund University (https://www.more-lab.lu.se/). This state-of-the-art facility for experimental health sciences features a reality platform, essentially an apartment designed as an instrumented home environment.

### Procedure

#### Sensor technology system

To capture detailed and accurate data for activity monitoring, we employed the Xsens MVN Awinda motion tracking system equipped with 17 inertial measurement units (IMUs), which were placed at key body locations. The Xsens system uses a network of IMUs strategically placed on the body to record motion in three-dimensional space. With high precision, these IMUs capture various aspects of human movement, including angular velocity, acceleration, and orientation^32^. This data is the foundation for analysing activity patterns and understanding human motion dynamics. We chose body-worn IMUs because they do not depend on lighting conditions, are robust to occlusions, and can capture detailed kinematic information, making them suitable for monitoring everyday activities in home environments.

The apartment inside MoRe-Lab had devices, including video cameras and microphones. The primary purpose of video cameras and microphones was to provide ground truth of the activities performed by the participants during the lab sessions. In the apartment, 5–7 video cameras are placed in each room except the bathroom. Cameras were strategically positioned to record the participants’ activities from multiple angles. The video footage was used to annotate the sensor data manually, associating each recorded motion sequence with the corresponding activity label. Video annotations also helped mitigate potential sensor noise or signal ambiguity issues, offering an additional validation layer to enhance the dataset’s quality. Combining the Xsens motion tracking system and video recordings provided a robust and complementary approach to capture activity data. This dual approach enhanced the dataset’s interpretability and ensured it could be used effectively for activity recognition.

#### Everyday activities

The data collected were based on the performance of everyday activities (Table 2), which are important to sustain independence^33^. The selection of activities was guided by commonly recognised activities of daily living (ADLs) and instrumental activities of daily living (IADLs), as well as their relevance to functional independence in older adults^33^. Although no formal standardised clinical assessment tool was strictly followed, the activity set was informed by domain knowledge within health sciences and aligned with clinical perspectives on functional ability. Each activity was selected to reflect common everyday activities, ensuring the data captured would represent natural behaviour.

**Table 2:** Everyday activities included in the study.

Activity	Description
1. Reading a newspaper	Reading two articles in a magazine that covers several pages
2. Conversation via mobile phone	Calling the facilitator for small talks and instructions for the next activity
3. Taking medication	Taking chocolate or lemon candy (as medicine) in a plastic bottle container and drinking water afterwards
4. Making coffee	Brewing coffee with a smart coffee maker using coffee powder
5. Preparing food	Simulating toasting bread in a pan, flipping the bread, and toasting both sides. A sandwich was prepared afterwards.
6. Eating and drinking	Eating the prepared sandwich with a knife and fork and drinking coffee
7. Washing dishes	Washing used dishes and utensils either by hand or putting them into the dishwasher.
8. Vacuuming	Simulating vacuuming open spaces and under furniture
9. Using the toilet	Simulating using the toilet without taking off clothes
10. Handwashing	Handwashing after a toilet visit
11. Putting on shoes	Simulating putting on shoes before outdoor walking
12. Walking outdoors	Walking around the outdoor premises of the apartment
13. Taking off shoes	Simulating taking off shoes after outdoor walking
14. Resting on the bed	Simulating resting on bed

#### Trial sessions

We conducted two trial sessions to test the wearables’ data-capturing capability, battery life, comfort, and restrictions in performing the 14 everyday activities. The trial sessions provided a better understanding of the sensors’ limitations in specific areas of MoRe-Lab and adjusted the data collection time for participants to allow a more natural execution of the activities.

During the trial sessions, wireless communication issues arose when participants were positioned in certain parts of the apartment. These issues were primarily attributed to signal interference and weak connectivity between the wearable devices and the central recording system. Areas near metallic objects or where wireless signals were weaker caused occasional data transmission delays or packet losses, which led to minor gaps in the recorded data. This highlighted the importance of optimising base station placement and ensuring consistent connectivity throughout the room to minimise data discrepancies.

The findings from these trial sessions allowed us to refine the experimental setup, such as repositioning the base stations and avoiding problematic areas, ultimately ensuring better data collection and reliability during the main study.

#### Data collection

The data collection process began with a detailed explanation of the activities to ensure that participants understood each task. This was followed by a tour through MoRe-Lab and a demonstration of the necessary household objects to perform the activity. The participants were asked to perform the activities at their own pace as they would naturally do in their own homes, which led to variable activity durations. Following this briefing, we carefully placed 17 IMUs using Velcro straps, as provided with the Awinda system, at key body locations on the participants, including the head, torso, arms, legs, and other joints crucial for capturing comprehensive motion data (Fig. 2). This placement was essential for accurate movement tracking across all major body segments. We then verified that each sensor had proper attachment and calibration, ensuring optimal functionality and minimal participant discomfort before performing the activities.

The 14 activities were then performed in the fixed order listed in Table 2 to optimise transitions between each activity, lasting 30–90 min for each participant. All participants used their right hand as their dominant hand when performing the activities. The data collection was facilitated by authors (WSG and NM), who also supervised the activities and interactions between the participants and the sensor systems and guided participants through each step. The facilitators maintained a supportive and attentive presence, ready to address questions or clarify instructions as required. Simultaneously, WSG recorded the data using video cameras, and NM recorded the sensor data. WSG and NM closely monitored the recording to ensure the cameras functioned correctly and that the recorded data was transmitted without interruption or interference. The participant’s data were logged and continuously stored during the session.

### Data analysis

#### Data labelling

Data labelling began by aligning the motion tracking data with real-world actions, providing a clear framework for subsequent analysis. We synchronised the recorded motion-tracking data with the camera feeds by asking each participant to perform a hand wave at the beginning and end of the recording session. The time point at which the hand reached the upward position was identified in both the IMU data using the avatar and the video streams, and used as a common marker to align the timelines.

We then annotated the video recordings captured during the participants’ activities. Each video was carefully reviewed, and timestamps corresponding to the beginning and end of each activity were identified. These timestamps were cross-referenced with the sensor data to label segments according to each activity, ensuring the accuracy and usability of the collected data. The annotation process involved meticulously marking the data to reflect transitions between activities (e.g., moving from sitting to standing or walking to turning) and identifying any deviations or interruptions during the activities.

For privacy reasons, no cameras were installed in the bathroom. Activities using the toilet and handwashing were annotated based on entering and exiting the bathroom, and by monitoring the corresponding sensor avatar (available within Xsens software) to detect sitting posture, standing posture, and handwashing posture.*Data preprocessing*.

Within the DL framework, selecting specific sensors (e.g., sensor placed on the right forearm, head) and the physical values (e.g., linear acceleration in the x-axis, orientation) associated with each sensor was possible. Only these columns were imported from the motion-tracking data. The training–testing split was performed in a leave-one-subject-out manner. The data from nine participants were used for training, and data from the remaining participant were used exclusively as the test set^34^. For early stopping, a validation subset was created by randomly partitioning the training data (from the nine participants) into 80% training and 20% validation. Thus, data from each participant never appeared simultaneously in the training and test sets.

Four common time-domain signal features were computed for each sensor signal: root mean square, mean absolute value, variance, and signal maximum within a sliding window^35^. The sliding window size was set to 200 samples (~ 3.3 s) and the stride to 60 samples (1 s). These values were empirically chosen during a small-scale parameter tuning process. During this procedure, we varied the window size (ranging from 5 to 200 samples) in a few steps, using only the model with five sensors (Model 1) to observe trends in classification accuracy. The upper limit value was set to keep the model size within the available graphics card memory. The tests indicated that using the window size of 100 samples resulted in the highest accuracy; therefore, for full-scale testing, a value of 100 samples was chosen (Table 3). The stride was selected with memory constraints in mind. Increasing the stride reduces the length of the input data; hence, in the final train-test setup, a stride of 20 was used, effectively reducing memory usage by a factor of 60 compared to the raw motion-tracking data.

**Table 3:** Model 1 with different window sizes.

Window size	5	20	50	100	200
Median accuracy (%)	80,4	82,4	81,6	86,1	83,6

The extracted feature signals were then prepared for input into the long short-term memory (LSTM) network, formatted as an (F*S)xLxH matrix, where F denotes the number of features (4 in this case), S the number of selected motion-tracking signals, L the length of the input data, and H the history of the input data supplied to the LSTM as delayed states^25,36,37^.

#### LSTM model

Since everyday activities consist of combinations of dynamic, cyclic, and static movements, it is essential to capture the kinematic signals over a specific time window. This approach accounts for variability in how a person performs an activity, including brief stationary moments. To handle time-domain series, we found that an LSTM network was an optimal solution; thus, we focused our analysis on this architecture^37^. The LSTM parameters, namely, the sequence length (input history) and the number of units, were optimised using the same procedure applied to tuning the sliding window size, stride, and sensor selection^37^. Based on the optimisation process, the LSTM size was set to 256 units and the input history to 15. With a stride of 60 samples, this configuration provided a total time memory of 900 samples (60 samples × 15 timesteps), corresponding to 15 s at a 60 Hz sampling frequency.

**Fig. 1:**
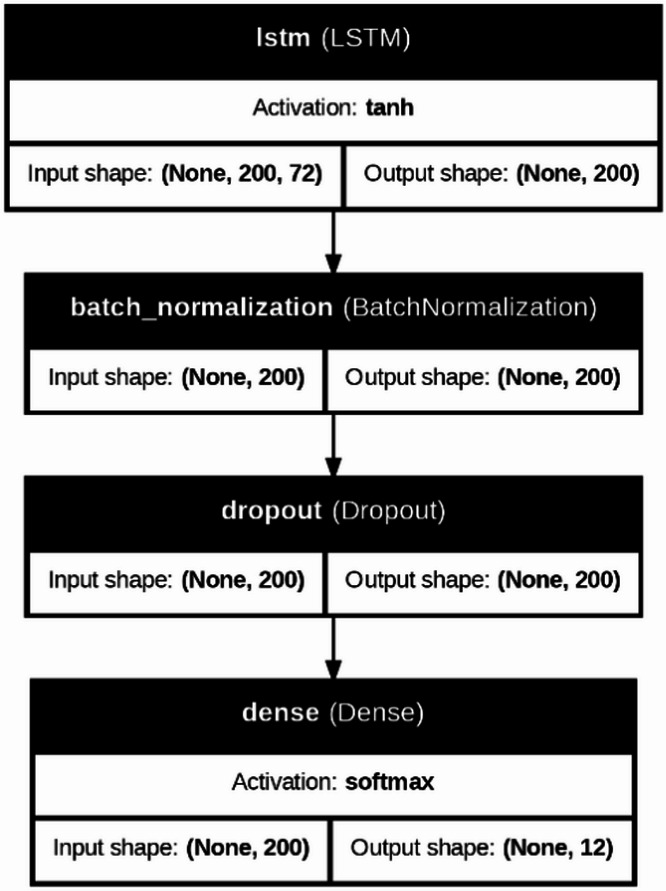
Architecture of the deep learning method used in this study. The sequence of the subset of kinematic signals was used as the input to the LSTM network, followed by a Batch normalisation layer, a dropout layer, and a fully connected (Dense) layer with the same number of outputs as the number of activities. The input size shown here is for Model 3: 2 sensors (right hand, pelvis) x 3 axis (x, y, and z) x 3 physical values (segment acceleration, segment orientation in Euler angle, segment angular velocity) x 4 features (RMS, MAV, variance and max). The output is 12 activities that were used for training/testing of this model.

Following the LSTM layer, we applied batch normalisation, then a dropout layer with a dropout rate of 0.2, and finally a fully connected layer with a softmax activation function (Fig. 1). For training, we used the RMSprop with the sparse categorical cross-entropy loss function^38,39^. Early stopping was implemented to reduce overfitting with a minimum delta of 0.0005 and a patience of 5 epochs. The validation subset used for early stopping was created by splitting the training data in an 80 − 20 ratio, with 20% reserved for validation.

To identify the most appropriate optimisation strategy, Model M1 with a window size of 100 was trained using several optimisers, namely Adam, RMSprop, Adadelta, Lion, and stochastic gradient descent (SGD). The median classification accuracies obtained with each optimiser are summarised in Table 4. Among all evaluated methods, RMSprop achieved the highest median accuracy (88.1%), outperforming Adam (86.1%) and all other alternatives. While Adam exhibited stable training behaviour, its performance remained consistently below that of RMSprop. Adadelta resulted in substantially degraded accuracy, whereas Lion and SGD also underperformed relative to the two leading optimisers. Based on its superior median accuracy together with reliable convergence behaviour across runs, RMSprop was selected as the optimiser for all subsequent models and analyses.

**Table 4:** Model 1 with a window size of 100 and different optimisers.

Optimiser	Adam	RMSprop	AdaDelta	Lion	SGD
Median accuracy (%)	86,1	88,1	38,3	83,3	78,9

The LSTM model in this study is relatively compact, comprising 308,014 trainable parameters with a model size of approximately 1.18 MB, and demonstrates efficient performance with an inference time of approximately 2–3 s per evaluation batch and training time of 320–550 s per fold.The dominant computational cost arises from the data preparation stage, including loading, segmentation, and feature extraction, which required approximately 1500 s per fold. The processed input data required about 4.9 GB of GPU memory, with memory usage scaling linearly with the number of extracted features and temporal modelling parameters such as stride and sequence length. Therefore, restricting the feature set to a small number of time-domain features was necessary to ensure computational efficiency and maintain feasibility within hardware constraints while still allowing sufficiently long temporal sequences.

The signal processing framework was implemented in Python 3.7, using libraries such as numpy (version: 1.26.4), scipy (1.14.1), matplotlib (3.9.2), pandas (2.2.3), keras (3.6.0), and sklearn (1.5.1). The hardware used in this study was PC with AMD Ryzen 7 PRO 4750G CPU, 128 GB of RAM memory and NVIDIA GeForce RTX 3090 GPU.

#### Analysis framework

We conducted a detailed performance analysis of the models using confusion matrices (Supplementary 1 Confusion Matrices). The confusion matrix offers a granular breakdown of the model’s classification results, displaying the counts of true positives, true negatives, false positives, and false negatives^40^. This detailed view highlights the areas where the model performs well and helps identify specific misclassification patterns^40^. We also perform participant-level performance scores across measurement points for all participants (Supplementary 2 Subject-level Performance Results).

To evaluate the performance of our DL model, we employed a comprehensive set of metrics and diagnostic tools that are widely recognised in the field^35,40^. Our overall performance assessment began with basic metrics such as accuracy, which measures the proportion of correct predictions relative to the total number of predictions made. However, since accuracy alone can be misleading, especially in cases of class imbalance, we further incorporated precision and F1-score into our evaluation.

Precision provides insight into the quality of positive predictions by indicating the ratio of true positive outcomes to all instances classified as positive. This metric is particularly useful when the cost of false positives is high. F1-score was added to evaluate the performance of a classification model in dealing with imbalanced datasets. By analysing precision and F1-score, we could understand the trade-offs between the model’s sensitivity and specificity. Finally, we used the Friedman test and the Wilcoxon signed-rank tests to evaluate the performance of the models. This test assessed whether the observed differences in accuracy, precision, and F1 scores were statistically significant^41,42^. Although we evaluated fifty-six classification models, each with approximately 6 h of running time, we only presented the results of five to satisfy the interest of this study.

The sensor combinations for the classification models were determined through an iterative process guided by domain knowledge (prioritising upper and lower limb segments and the pelvis) and empirical performance. We first considered baseline configurations (e.g. single forearm sensor resembling smartwatch placement), and then incrementally added or removed sensor locations based on their impact on validation performance and practical considerations (wearability, obtrusiveness).

#### Survey data

We also collected data on the participants’ experience using the sensor technology through a short questionnaire, inspired by the Technology Acceptance Model^43^. A descriptive analysis was conducted to summarise participant responses^44^. Frequencies were used to highlight variation in the experiences and perspectives of using wearable sensors.

## Results

Five LSTM-based models were evaluated, summarising their configurations and performance metrics in Table 5. As shown in Table 5, the models varied in terms of the number of sensors used, the number of activities classified, key hyperparameters (e.g., window size, stride, and LSTM units), and performance. Model 0 was chosen as the reference model as it is based on a single IMU sensor placed on the forearm, roughly comparable to the placement of commercial wrist-worn devices. The other models (Models 1–4) were selected as the best performing among fifty-six models tested using various parameters and sensor combinations.

**Table 5:** Parameter combinations, accuracy, and recognition performance of the five representative models.

Parameters	Model 0	Model 1	Model 2	Model 3	Model 4
LSTM units	200	200	200	200	200
Window size	100	100	200	200	200
Time steps	200	150	200	200	200
Stride	60	60	60	60	60
Sensor signals (IMU locations)	1 Right Forearm	5 Right Forearm Head Right Hand Right Upper Leg Left Upper Leg	7 Right Forearm Left Forearm Head Right Hand Left Hand Right Upper Leg Left Upper Leg	2 *Pelvis* *Right Hand*	3 *Pelvis* *Right Hand* *Head*
Number of activities	14	14	12	12	12
Reading a newspaper	0	25.6	0	0	0
Conversation via mobile Phone	0	1.2	x	x	x
Taking medication	0	2.6	x	x	x
Making coffee	9.5	79	93.4	68.3	41.5
Preparing food	0	99.9	99.4	95.9	97.7
Eating and drinking	25.1	99.7	98.8	92.8	89.1
Washing dishes	91.6	96.4	92.5	82.9	85.4
Vacuuming	69.5	98.7	93.6	93.9	96.4
Using the toilet	40	90.7	90.2	72.7	75.9
Handwashing	90.8	88.7	92.4	71.7	90.8
Putting on shoes	26.4	90.9	97.2	88.6	66.7
Walking outdoors	58.5	99.5	100	97.6	98.5
Taking off shoes	17.7	85.4	64.4	87.8	88.3
Resting on bed	62.1	99.9	99.8	98.8	99.3
Accuracy	66.2%	88.2%	86.0%	89.3%	84.3%
Precision	73.1%	90.3%	89.0%	90.1%	88.3%
F1 Score	68.8%	86.1%	84.9%	89.4%	84.7%

### Sensor configuration and activity recognition

The results indicate a clear relationship between the number and placement of sensors and the model’s performance.

Model 0 served as the baseline, which utilised a single sensor on the right forearm, performed poorly across all performance metrics (Accuracy: 66.2%; F1 Score: 68.8%). This minimal setup was only able to detect a limited subset of activities and lacked the sensitivity to capture more complex or full-body actions. This may suggest that relying on a single sensor limits the model’s ability to distinguish between complex or overlapping everyday activities.

Model 1, which used five sensors (right forearm, head, right hand, right upper leg, left upper leg), showed a marked improvement in recognition performance (Accuracy: 88.2%; F1 Score: 86.1%). It recognised all 14 activities, demonstrating that multiple sensor points, especially covering both upper and lower extremities, significantly enhance recognition capability.

Model 2, with seven sensors (including both left and right limbs and the head), achieved an overall performance (Accuracy: 86.0%; F1 Score: 84.9%). This comprehensive configuration enabled detailed motion capture, resulting in highly reliable recognition of more complex activities, such as preparing food, using the toilet, and vacuuming.

Model 3 shows that strong performance can be achieved with just two sensors. Despite recognising two fewer activities than Models 0 and 1, Model 3 maintained high classification quality, demonstrating a promising balance between practicality and effectiveness.

Model 4 added a head sensor to Model 3’s configuration. This provided a slight decrease in precision and did not improve the F1 score, suggesting diminishing returns when adding more sensors to already efficient minimal configurations.

**Fig. 2:**
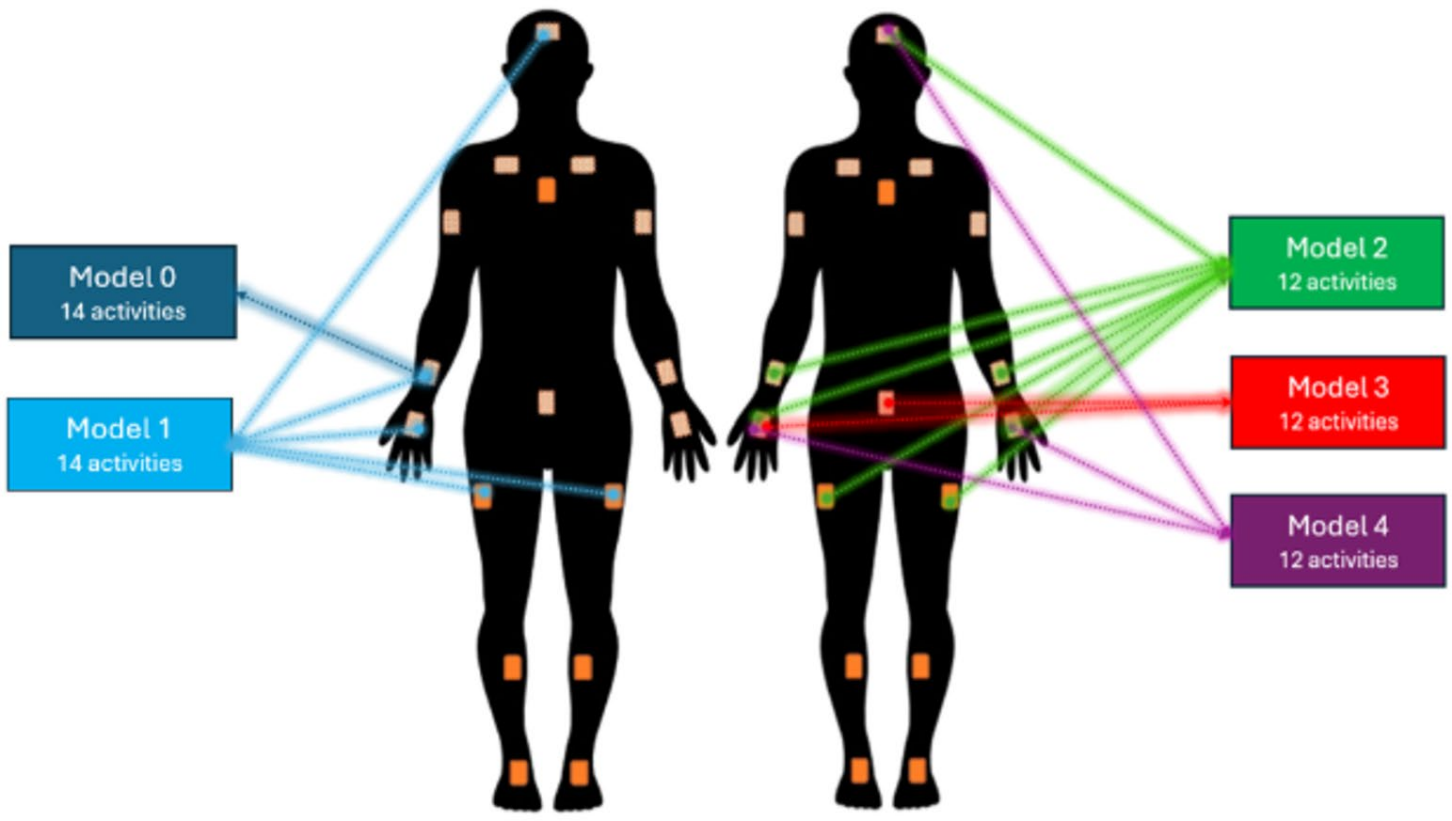
Sensor placements and activity recognition scope across five LSTM-based models. Each model utilises a different combination of body-worn inertial sensors (represented by orange squares) connected via colour-coded lines to the corresponding model (Model 0 to Model 4). Adapted from Xsens motion capture technology.

The evaluation of the five models highlights insights into sensor-based activity recognition using LSTM networks. Figure 2 illustrates the distribution of sensor positions on the body (e.g., hand, forearm, pelvis, head, legs) and the number of everyday activities recognised by each model.

Although the Xsens system provides motion data from 17 IMUs, we did not use all sensors in every model. Our focus was to explore the extent to which the sensor configuration could be reduced while preserving model performance.

### Cross validation

The five models reveal differences in performance, emphasising how sensor placement and the activities affect activity recognition. Model 3 outperforms the others across all key metrics, with the highest classification accuracy. This suggests that Model 3 not only classifies activities more accurately but also does so consistently across multiple trials. The performance, characterised by high precision and an F1 Score, can be attributed to optimised sensor positioning and improved hyperparameter techniques. In contrast, Models 1, 2, and 4 exhibit slightly lower accuracy, indicating greater variability in their ability to correctly classify different activities.

Models 3 and 4 highlight the trade-offs between sensor complexity and model performance. Although Model 4 introduces an additional sensor (head), it does not yield a performance improvement across key metrics. Model 3 demonstrates a more favourable balance with fewer sensors, while still achieving high accuracy, precision, and F1 score. These metrics suggest that Model 3 can reliably detect everyday activities included in this study.

**Fig. 3:**
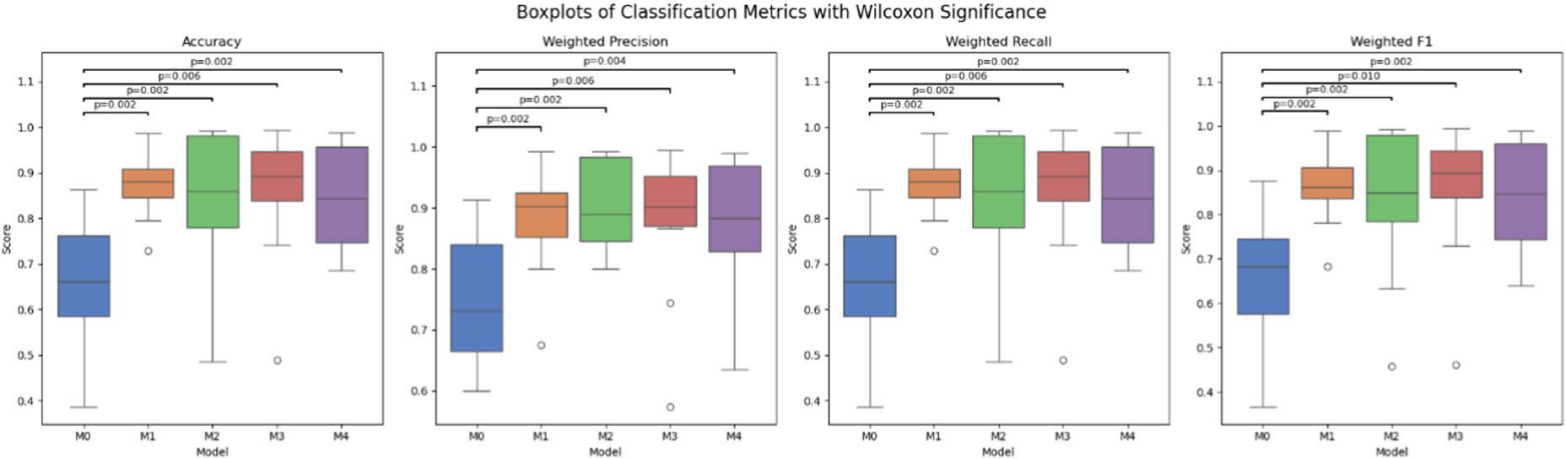
Boxplot of classification metrics for Models 0 to 4. The chart illustrates the distribution of accuracy, weighted precision, weighted recall, and weighted F1-scores across five activity recognition models for their overall performance and variability.

In evaluating the performance of Models 0 through 4, a Friedman test (followed by Wilcoxon signed-rank tests) was conducted to assess whether the observed differences in accuracy, precision, and F1 scores were statistically significant (Fig. 3). The results indicated statistically significant differences between the performance metrics of Model 0 and Models 1–4. This highlights the importance of incorporating additional and diverse sensor data. On the other hand, the results also demonstrated no statistically significant differences in the performance of Models 1 through 4. This is visually and numerically supported by the close range and overlapping values of each model’s scores. For instance, although Model 3 exhibited slightly higher performance across all metrics, achieving an F1 score of 89.4%, and Model 4 showed somewhat lower values (e.g., 84.3% accuracy), these variations were not significant enough to distinguish one model as superior to the others in a statistical sense.

This suggests that Models 1 to 4 can be functionally comparable in their ability to recognise and classify everyday activities. From a practical perspective, this means that the choice between models may depend less on predictive performance and more on contextual considerations such as the number and placement of sensors, user comfort, cost, and ease of deployment.

**Fig. 4:**
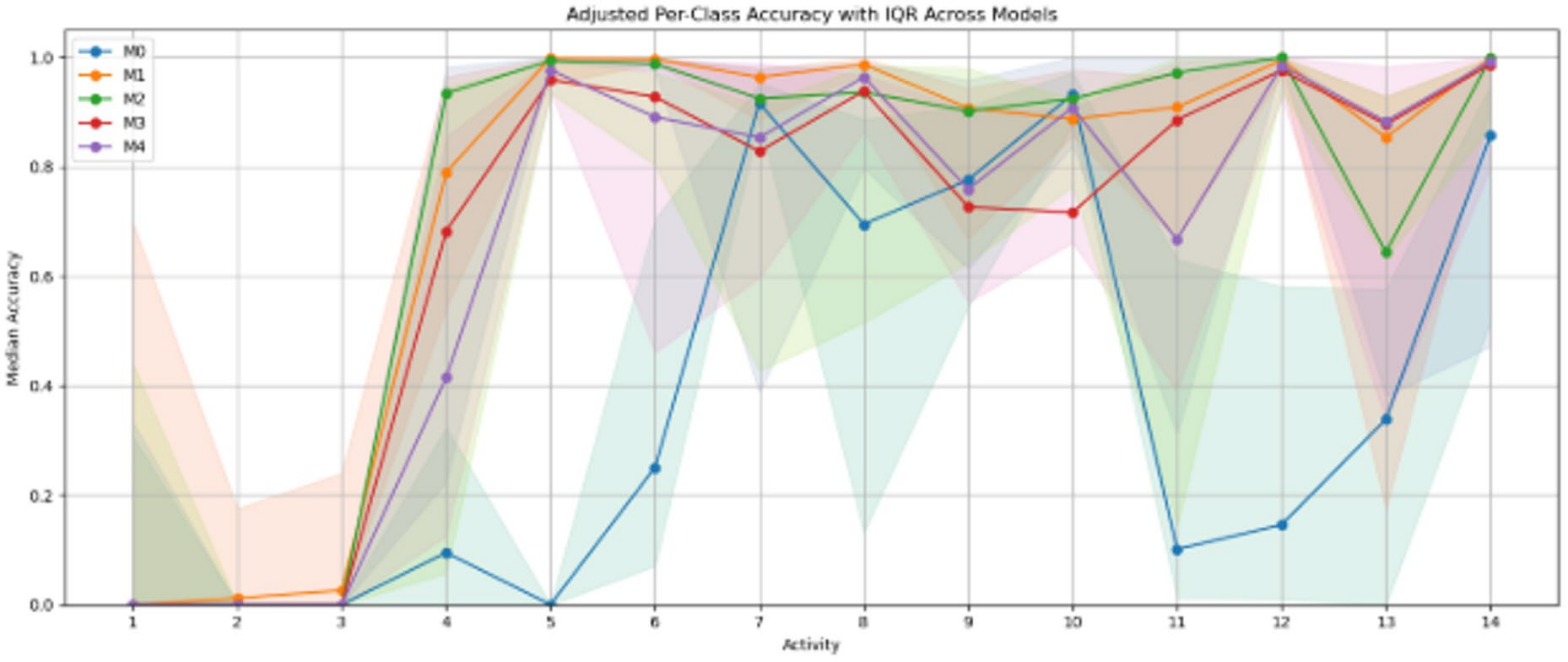
Adjusted per-class accuracy with interquartile range (IQR) across models 0 to 4. The figure illustrates each model’s median per-class accuracy and variability (IQR shading), highlighting performance patterns across different activity classes.

Figure 4 presents the adjusted per-class accuracy for the five models, highlighting differences in their ability to recognise various activities. Across all models, accuracy is low for the first three activities ((0) reading a newspaper, (1) conversation via mobile phone, and (2) taking medication), suggesting that these activities are inherently harder to detect, possibly due to subtle or overlapping movements. The narrow interquartile ranges (IQRs) in these classes indicate that this difficulty is consistent across participants. From Class 3 onwards, all models show a sharp improvement, with median accuracies rising above 0.8, suggesting these activities are easier to distinguish. Among the models, model 2 consistently achieves the highest accuracy and demonstrates more stable performance. In contrast, models 3 and 4, with fewer sensors, show greater variability, particularly in classes 8 and 10, where accuracies dip, and IQRs widen. Again, this indicates that a reduced sensor configuration limits the models’ ability to reliably classify more complex or similar activities.

Model 0 underperforms across most classes. Overall, while Model 2 offers the best performance across the board, the differences among Models 1–4 are relatively modest, with no statistical significance. This highlights that model selection may be guided less by marginal performance differences and more by practical considerations such as sensor burden, user comfort, and implementation cost.

To provide a clearer performance context, we have extended the study by including three widely used baseline classifiers: Logistic Regression (LR), Random Forest (RF), and Support Vector Machine (SVM). The benchmarking results (using the Wilcoxon signed-rank test for paired samples, α = 0.05) demonstrate that the LSTM-based models consistently outperform traditional machine learning approaches across all configurations. The results indicate that the performance improvements of the LSTM model are statistically significant in all comparisons, except for the M2 LSTM vs. Random Forest comparison, where the difference was not statistically significant (*p* = 0.08). As shown in Table 6, LSTM achieved the highest accuracy for every model, with performance ranging from 66.2% (M0) to 89.3% (M3), while LR, RF, and SVM showed substantially lower accuracy. RF performed best overall but still remained below the LSTM results in all cases. Furthermore, we have added a table comparing LSTM, LR, RF, and SVM across training speed, prediction speed, and model complexity (Supplementary 3 Comparison of Computational Performance).

**Table 6:** Comparison of classification accuracy across models (M0–M4) using LSTM and traditional machine learning algorithms (LR, RF, SVM), showing consistently superior performance of the LSTM-based approach.

Models	Algorithm accuracy [%]			
	LSTM	LR	RF	SVM
M0	66,2	33,4	52,8	27,0
M1	88,1	67,2	73,3	41,1
M2	86,0	70,9	79,0	44,3
M3	89,3	53,6	76,4	32,0
M4	84,3	59,3	77,1	31,8

### Participants’ experience of the sensors

The survey results indicate that 8 out of 10 participants did not experience the sensors as intrusive or disruptive, with minimal concerns about privacy and surveillance. Half the participants expressed interest in using sensors to track their everyday activities, reinforcing their potential value. 10 out of 10 participants reported that the presence of sensors would not hinder their everyday activities at home or outdoors. This suggests a general acceptance of wearable sensors in everyday routines. Additionally, 6 out of 10 participants expressed a positive attitude toward their experience with the sensors. However, the remaining participants showed either neutral or negative responses, highlighting the need for further studies to fully understand varying comfort levels and expectations. Some participants also reported discomfort with wearable devices and dissatisfaction with the aesthetics of the sensors. 7 out of 10 participants noted that the appearance or physical design of the sensors was unappealing.

## Discussion

Our study presents a home-lab dataset of 14 everyday activities performed by older adults using a full-body IMU system. We explore sensor configurations and demonstrate that a two-sensor configuration (pelvis and wrist) can recognise 12 activities with high accuracy. Our results combine quantitative performance analysis with participants’ experiences of using wearable sensors, highlighting acceptability and design considerations for real-world deployment. More importantly, the inclusion of older adults in performing everyday activities contributes to the dataset to train a recognition model.

### Sensor placement and quantity

Our results show that increasing the number of sensors generally improves classification accuracy, as evidenced by Model 2 achieving an accuracy of 86.0% when using seven sensors. This model also includes bilateral forearms and legs, allowing for symmetrical body movement capture. However, it is not solely the number of sensors that matters, optimal sensor positioning also plays a critical role. For instance, Model 3 used two sensors to classify 12 activities and achieved 89.3% accuracy. Model 2 and Model 3 have classified the same number of activities and differ in sensor signal count, but yield a comparative accuracy.

Moreover, configurations with fewer sensors (as in Models 3 and 4, which used 2 and 3 sensors) produced competitive results. These models suggest that a minimal sensor setup can still capture the essential movement patterns needed for accurate activity recognition when redundant or less informative sensor locations are removed.

It is also worth noting that the selection of sensors for all five models was largely driven by intuition, experience, and an iterative trial-and-error approach. This method enabled progressive refinement of sensor placement based on initial experimental outcomes, ultimately balancing performance with practical considerations such as user comfort and system complexity.

### The impact of the number of activities on accuracy

The number of activities being classified in our models directly influences accuracy, with a higher number of activities generally leading to greater classification difficulty and lower accuracy. For example, Models 0 and 1, which attempted to distinguish 14 activities, achieved a lower accuracy compared to other models. In contrast, all other models classified 12 activities and reached a higher accuracy. Interestingly, with only two sensors, Model 3 recognises 12 activities, but still maintains a relatively high accuracy. This result suggests that a well-equipped sensor setup can mitigate the complexity introduced by additional activities.

Our findings reveal that recognition accuracy tends to decline as the number of activities increases, a challenge also noted in prior work^20,45^. One key factor is class overlap, where activities share similar motion patterns, making it difficult for models to distinguish between them. Activity duration further complicates model performance. Data imbalance with short-duration activities provides limited training data, reducing the model’s exposure to intra-class variability and increasing the risk of underfitting^46^. In contrast, longer-duration activities may contribute more training samples and richer temporal features. Yet, when these longer activities closely resemble shorter ones, they may introduce bias. For instance, eating and drinking may be overrepresented and share features with shorter actions like taking medicine, leading the model to favour the more dominant class. This highlights a broader challenge in real-world activity recognition to deal with the balance between activity diversity, class distinctiveness, and equitable representation across classes, particularly when aiming to scale systems for real-time use in complex environments^47^.

### What sensor combination to choose?

In this study, we also explored the one-sensor approach in the iterative process of our analysis, which shows a low recognition performance. The one-sensor approach underperforms consistently across all metrics. It has lower median scores and higher variability, especially in weighted recall and weighted F1-score. This suggests it may not be a reliable classification model for 14 or 12 everyday activities. However, Awais et al.^47^ and Meng et al.^48^ argued that the performance of their proposed state-of-the-art single-sensor approach is close to that of multiple sensors.

Overall, Models 1 and 3 are the most accurate and consistent across all metrics, likely due to optimised sensor placement and advanced processing techniques. However, the decent precision of Model 3 highlights the impact of participant handedness, suggesting that sensor location on the dominant side may improve detection accuracy. These findings reinforce the importance of considering individual characteristics and sensor placement symmetry when designing recognition systems.

Based on the presented results, we proposed a two-sensor method (Model 3) to collect and correctly recognise everyday activities among older adults. Despite the encouraging performance of the 3-sensor approach (Model 4), its practical application of having more sensors can interfere with a person’s activities and maintenance issues. Also, adding more sensors increases computational complexity, energy consumption, and costs, which might not always be practical in real-world applications. Research on adopting wearable technologies among older adults consistently underscores the value of comfort, simplicity, and intuitiveness^49,50^. This is further supported by our results, which show that some participants reported discomfort with sensors and dissatisfaction with the design. The placement of sensors in Model 3, which is limited to the pelvis and right hand, represents a more discreet and less demanding configuration compared to Model 4, which requires a head-mounted sensor. In addition, the availability of head sensors is still underdeveloped, which can contribute to implementation challenges.

The marginal gain in precision seen in Model 4 does not compensate for the added complexity. High-precision detection is only one part of a larger ecosystem of acceptability, especially when wearable sensors are meant to operate seamlessly within the everyday lives of older adults. The goal is not only to achieve high technical performance but also to ensure that older adults are willing and able to use such technologies consistently and without frustration. On a societal level, systems based on models like Model 3 could enhance cost-effectiveness and scalability in public health and care of older adults. As governments and municipalities seek to integrate more welfare technologies into community-based care^51^, everyday activity monitoring that balances accuracy with ease of deployment and user comfort will likely gain greater traction.

It must be noted that the results obtained depend on the available experimental data used to train the classifiers and the everyday activities included. The dataset analysed in this study was collected in a home lab setting that may differ from other home settings. Participants were supervised but still able to perform their tasks freely, thus resulting in unbalanced data samples of everyday activities, where some activities were longer or shorter than others. These variations introduce inconsistencies in the data, making it harder for the model to establish recognition patterns that work across the participants. We, therefore, used the weighted metrics for less biased results. On the contrary, the unbalanced data samples truly reflect real-world conditions where the frequency and act of performing everyday activities cannot be controlled and supervised.

### Participants’ experience of the sensors

The participants’ experiences with the sensors used in this study provided insights into the practical usability and acceptability of the system in real-world contexts. Overall, responses from the post-trial questionnaire indicated a generally positive experience. Most participants found the sensors comfortable to wear for the short duration of the experiment. However, the concerns reported suggest that while the functionality may be acceptable, design plays a significant role in the use and home integration. This issue is consistent with our previous results, which can influence the decision to adopt technology^52^.

Many older adults may hesitate to use monitoring technology if they feel it compromises their independence or is too complex to operate. Some evidence found that involving older adults in the development process, such as design, significantly improves usability and trust in the technology, leading to increased adoption rates^53–55^. Ensuring sensor-based devices are discreet, comfortable, and easy to use can encourage widespread acceptance.

Another important consideration is the role of user-centred design in improving recognition models. Other studies show that incorporating participant-specific factors such as handedness, gait patterns, and activity preferences can enhance model performance and user satisfaction^56^. Personalised sensor configurations where sensors are positioned based on individual needs have been found to improve recognition accuracy and system usability, especially in healthcare applications where precise monitoring is essential. This suggests that future recognition systems should prioritise adaptive approaches that account for individual needs, preferences and variability^57^. In the current study, we did not develop personalised models. Our focus was on a single model generalising across participants to explore sensor configurations. However, Kumar et al.^57^ suggest that personalised or hybrid models could improve performance, particularly for activities influenced by individual movement patterns. Given the limited per-participant data in our study, we consider personalisation a key priority for future work. Future research must explore older adults’ perspectives on how everyday activities can become central to designing activity recognition systems. This can offer insights for creating value-driven, personalised activity monitoring that can adapt effectively to diverse user needs and resonate more with older adults’ routines.

### Limitations and strengths

All data were collected in the same physical environment, which is a potential limitation. Specifically, all data was collected in a lab setting designed to mimic a real home but still operate as a controlled environment. While this setup ensured consistency and control over external variables such as lighting, noise levels, and layout, it may not fully capture the diversity of real-world home environments. Variability in factors like home design, furniture arrangement, and background activity, which are common in natural settings, could potentially influence the performance of activity monitoring wearable sensors. As a result, the generalizability of the findings to actual, uncontrolled home environments may be limited. Future research should aim to validate these results in a diverse range of homes with different furniture configurations to assess the robustness and adaptability of the methods used. Also, the modest sample size limits generalisability.

A further limitation of this study is the lack of a systematic evaluation of all possible model combinations arising from 17 body-worn inertial sensors, 14 everyday activities, and multiple fine-tuning parameters. In principle, constructing models for each unique configuration of sensors, activities, and parameters (e.g., network size, window size) would provide a more comprehensive understanding of performance. However, with more than 130,000 potential models and each requiring about six hours of computation, such an undertaking would demand close to 90 years of processing time, making it unfeasible under current conditions. Future research should therefore focus on leveraging greater computational resources and more efficient optimisation techniques to enable such large-scale exploration within a realistic timeframe.

Another limitation of the study is the short duration of some activities, which limits the LSTM model’s ability to recognise such activities. While the short duration of some activities posed challenges for recognition accuracy due to imbalanced data, this limitation can also be seen as a strength of this study. By including brief, real-world activities, such as conversation via mobile phone or taking medicine, we were able to expose a critical limitation of current activity recognition models, which is their reduced ability to detect and recognise activities that occur over a short time span. This reflects the true complexity of everyday activities and demonstrates the need for models that are sensitive not only to prolonged activities but also to short-time span activities that are nonetheless essential for independent living, particularly among older adults. Future work should compare LSTMs with other state-of-the-art network architectures on the same dataset as benchmarks.

Furthermore, the strength of this study lies in the inclusion of older adults as participants, a demographic that is often underrepresented in activity recognition research. By involving older adults, this study enhances its ecological validity for real-world applications in healthcare and research. Additionally, we explored participants’ experiences with the wearable sensors through a brief questionnaire assessing factors such as design, privacy, comfort, usability, and willingness to continue using the sensor devices. This user-experience perspective offers valuable insights into the practical feasibility and acceptability of sensor-based monitoring, which are critical for long-term adoption and implementation.

## Conclusion

This study presents a method that can accurately recognise the everyday activities of older adults performed in a home setting. The analysis shows that 14 activities can be recognised with a decent overall model performance accuracy. With 12 activities included, the proposed two-sensor-based system enhanced model performance while utilising fewer sensors.

Another important contribution of this study is its potential to enable large-scale data collection. Because the proposed model only requires two wearable sensors (pelvis and dominant hand), it is more practical and acceptable for everyday use than having several sensors. This minimal configuration could therefore facilitate large-scale, long-term data collection on the everyday activities of older adults in their homes. Using this proposed method, data on everyday activities can be collected and monitored at home. Further development could lead to systems that can assess and monitor functioning. Home monitoring systems may enhance the quality of life and maintain the independence of older adults by promoting safety, timely intervention, and greater autonomy in everyday activities.

## Supplementary Information

Below is the link to the electronic supplementary material.

Supplementary Material 1

Supplementary Material 2

Supplementary Material 3

## Data Availability

The data generated and underlying results presented in the study contain sensitive information about the study participants, and they did not provide consent for public data sharing. Kinematics data (maybe even published in the near future) could be shared by request from a qualified academic investigator for the sole purpose of replicating the present study, provided the data transfer is in agreement with EU legislation on the General Data Protection Regulation and approval by the Swedish Ethical Review Authority. Contact information: Department of Health Sciences, Lund University, Box 117, 22100 Lund, Sweden. Principal investigator: Associate Professor Steven M. Schmidt, steven.schmidt@med.lu.se. The study reported in the manuscript was not preregistered.
