# Supplementary material for: Recognition of everyday activities using experiment data from wearable sensors: a deep learning-based framework
Source: Sci Rep. 2026 Jul 24;16:23218. doi: 10.1038/s41598-026-63774-8 (PMC13400646; doi:10.1038/s41598-026-63774-8)
Supplement: Supplementary file 1 — Supplementary Material 1 [file 41598_2026_63774_MOESM1_ESM.pdf]

Supplementary Table 1. Performance scores across measurement points (M0–M4) for subjects S1–S10, including descriptive statistics.

Subject	*M0*	*M1*	*M2*	*M3*	*M4*
S1	76,5	87,9	99,2	98,5	97,3
S2	77,1	91,6	97,4	92,7	98,8
S3	63,6	79,6	91,1	74,2	88,2
S4	86,4	98,7	98,5	99,5	98,8
S5	39,2	88,4	80,1	86,2	74,5
S6	57,0	72,9	69,1	49,0	68,6
S7	75,5	84,2	80,9	83,0	80,5
S8	68,8	98,1	98,5	95,3	91,3
S9	38,6	88,4	48,6	91,6	68,6
S10	62,8	86,1	77,3	87,0	75,3
Mean	64,5	87,6	84,1	85,7	84,2
Median	66,2	88,1	86,0	89,3	84,3
Std	15,1	7,4	15,6	14,2	11,6
Min	38,6	72,9	48,6	49,0	68,6
Max	86,4	98,7	99,2	99,5	98,8
