# Supplementary material for: Recognition of everyday activities using experiment data from wearable sensors: a deep learning-based framework
Source: Sci Rep. 2026 Jul 24;16:23218. doi: 10.1038/s41598-026-63774-8 (PMC13400646; doi:10.1038/s41598-026-63774-8)
Supplement: Supplementary file 2 — Supplementary Material 2 [file 41598_2026_63774_MOESM2_ESM.pdf]

Supplementary Figure 1 -5

Supplementary Figure 1-5 presents the row-normalised confusion matrix for Model 0 to Model 4, where each row corresponds to the true activity label and each column corresponds to the predicted activity label. Values represent the proportion of predictions for each true class, with rows summing to 1. The colour intensity indicates classification probability (darker blue = higher proportion). The models evaluate everyday activities, including reading a newspaper, calling someone, taking medicine, making coffee, preparing food, eating, washing dishes, vacuuming, using the toilet, handwashing, putting on shoes, walking outside, taking off shoes, and resting on the bed. The diagonal elements represent correct classifications, while off-diagonal values indicate misclassifications.

Model 0 (Supplementary Figure 1) shows strong performance across several activities, such as preparing food, vacuuming, walking outside, and resting on a bed, as evidenced by high diagonal values. However, noticeable confusion occurs among visually or motion-similar activities, such as reading a newspaper vs calling someone, making coffee vs preparing food, and hand-related hygiene and dressing activities, reflecting overlaps in motion patterns.

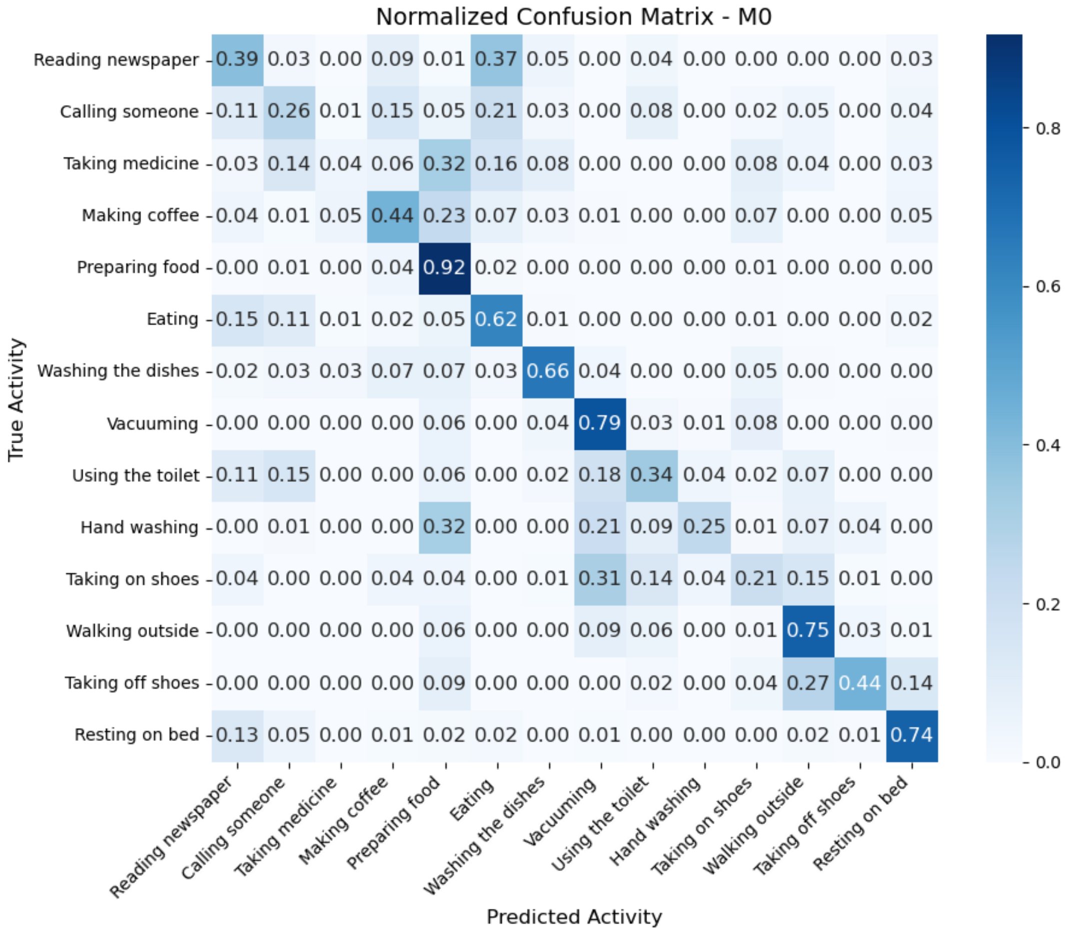

Supplementary Figure 1. Normalised confusion matrix for Model 0 (M0) across 14 everyday activities.

Model 1 (Supplementary Figure 2) performs well for activities with distinctive motion or location patterns such as eating, walking outside, and resting on bed. Lower performance for activities such as calling someone, taking medicine, and taking on shoes suggests overlapping sensor signatures or similar movement patterns with other daily activities. This indicates that fine-grained sedentary or transitional actions remain more difficult to discriminate than dynamic or context-specific tasks.

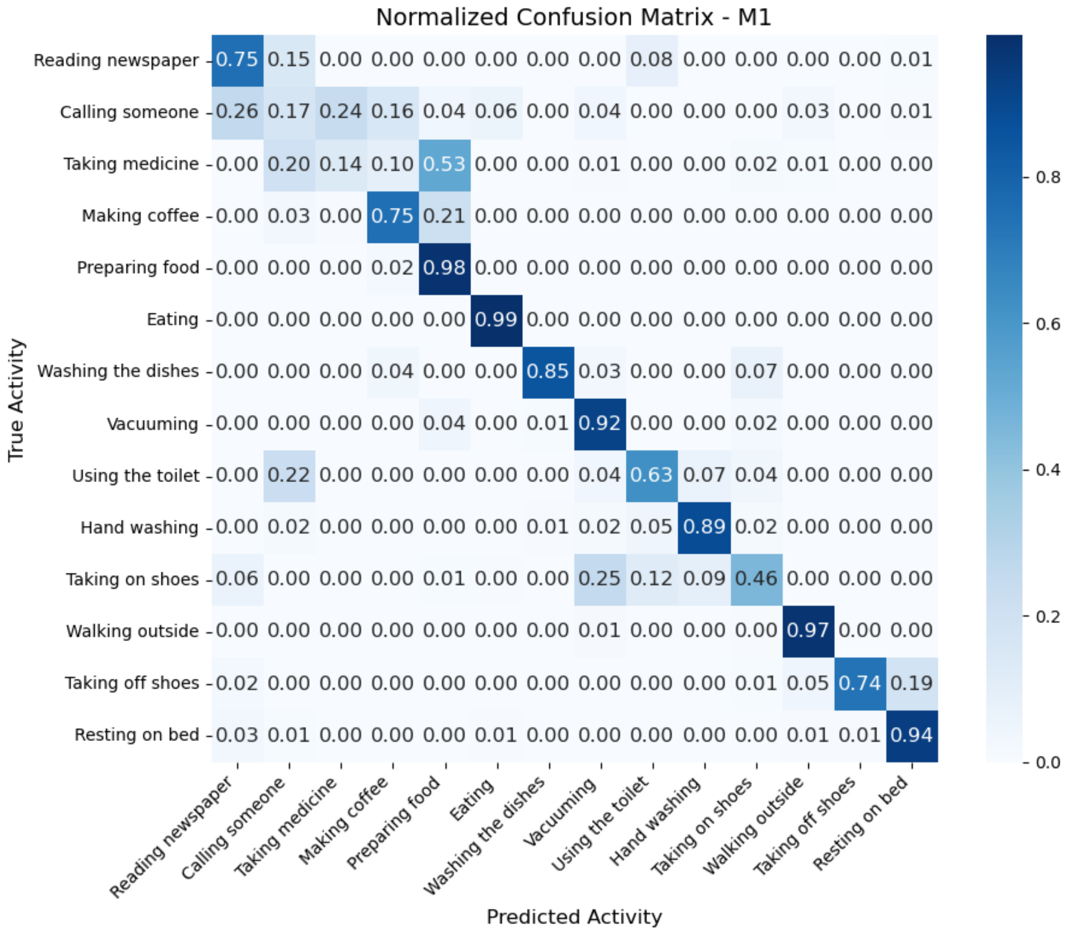

Supplementary Figure 2. Normalised confusion matrix for Model 1 (M1) across 14 everyday activities.

Model 2 (Supplementary Figure 3) demonstrates strong performance for activities involving clear movement or contextual patterns, such as preparing food, eating, walking outside, and Vacuuming. Moderate performance is observed for household and hygiene-related tasks. Lower recognition rates for reading newspaper and taking on shoes suggest difficulty distinguishing sedentary or transitional activities with overlapping behavioural signatures. Misclassifications between related household tasks (e.g., making coffee and preparing food) indicate shared motion patterns that may require richer contextual features for improved separation.

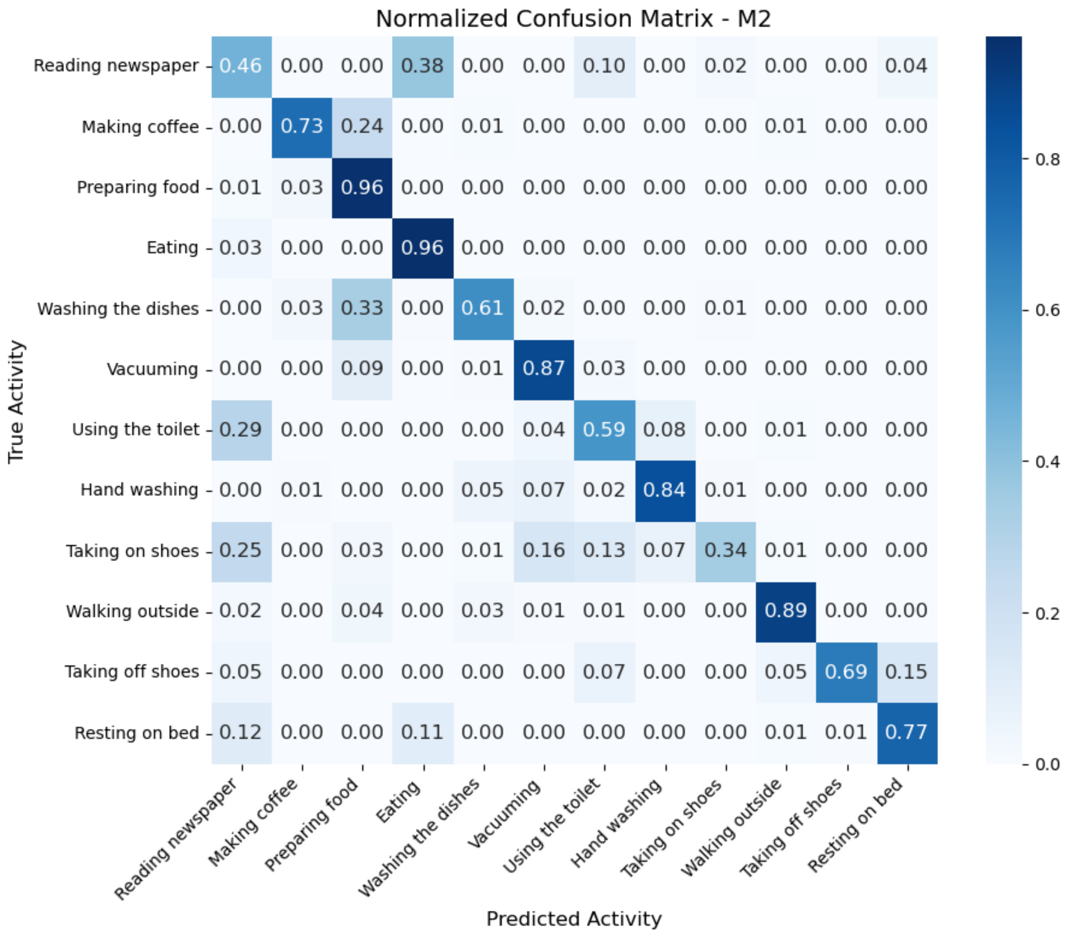

Supplementary Figure 3. Normalised confusion matrix for Model 2 (M2) across 12 everyday activities.

Model 3 (Supplementary Figure 4) demonstrates strong overall performance, particularly for activities with clear contextual or movement signatures such as preparing food, eating, vacuuming, walking outside, and resting on bed. Compared with earlier models, recognition of reading newspaper, using the toilet, and taking off shoes appears improved. However, taking on shoes remains the most challenging activity, likely due to its brief and transitional nature or overlap with other seated and household behaviours. Misclassification between kitchen-related activities (making coffee, preparing food, and washing the dishes) suggests shared motion patterns that remain difficult to fully separate.

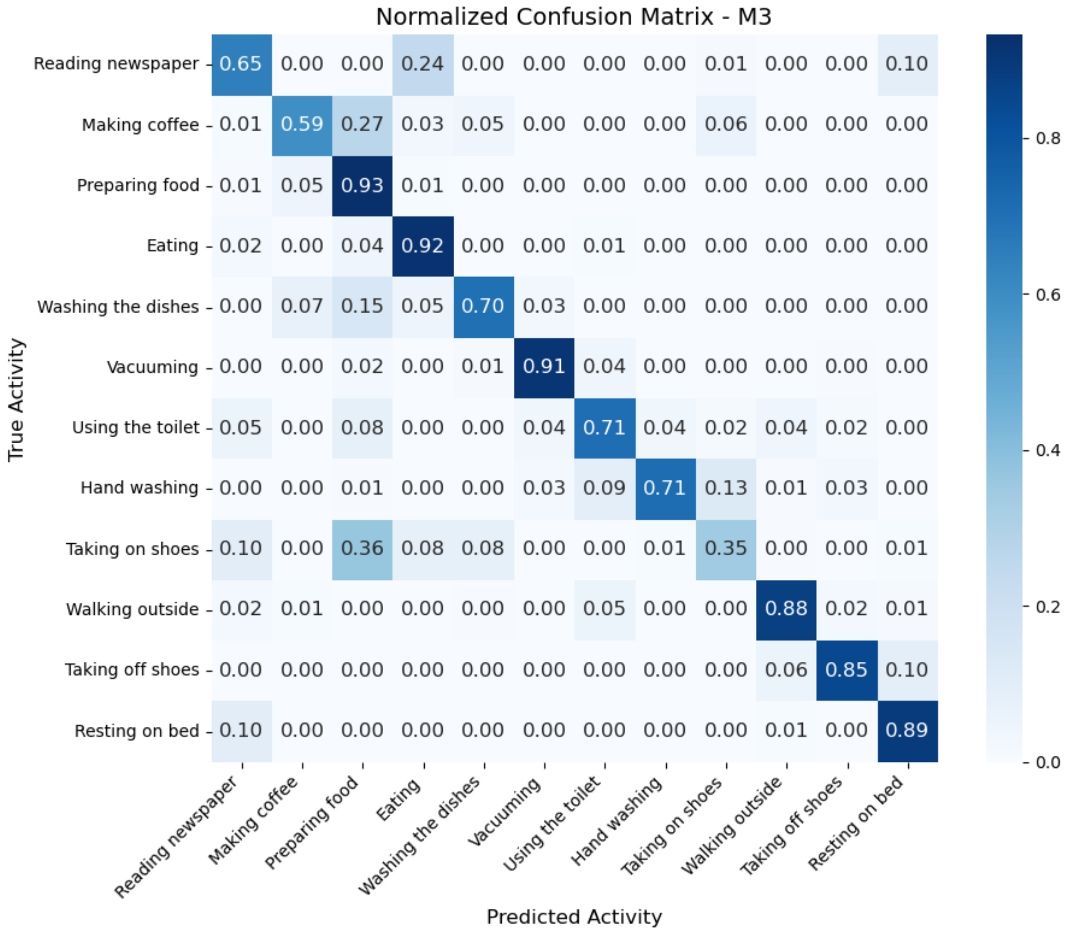

Supplementary Figure 4. Normalised confusion matrix for Model 3 (M3) across 12 everyday activities.

Model 4 (Supplementary Figure 5) demonstrates strong classification performance for most activities, with the highest recognition rates observed for resting on bed, preparing food, eating, walking outside, and taking off shoes. These high diagonal values indicate that the model can reliably distinguish activities with distinct motion patterns and environmental context. Significant confusion occurs between semantically related activities. For example, making coffee is frequently misclassified as preparing food and washing the dishes, while taking on shoes is often confused with Vacuuming, using the toilet, and Hand washing. Similarly, using the toilet shows confusion with preparing food and walking outside. These misclassifications indicate overlapping behavioural patterns and similar contextual sensor signatures. The confusion matrix confirms that Model 4 performs well on clearly distinguishable activities but struggles to recognise transitional or contextually similar actions.

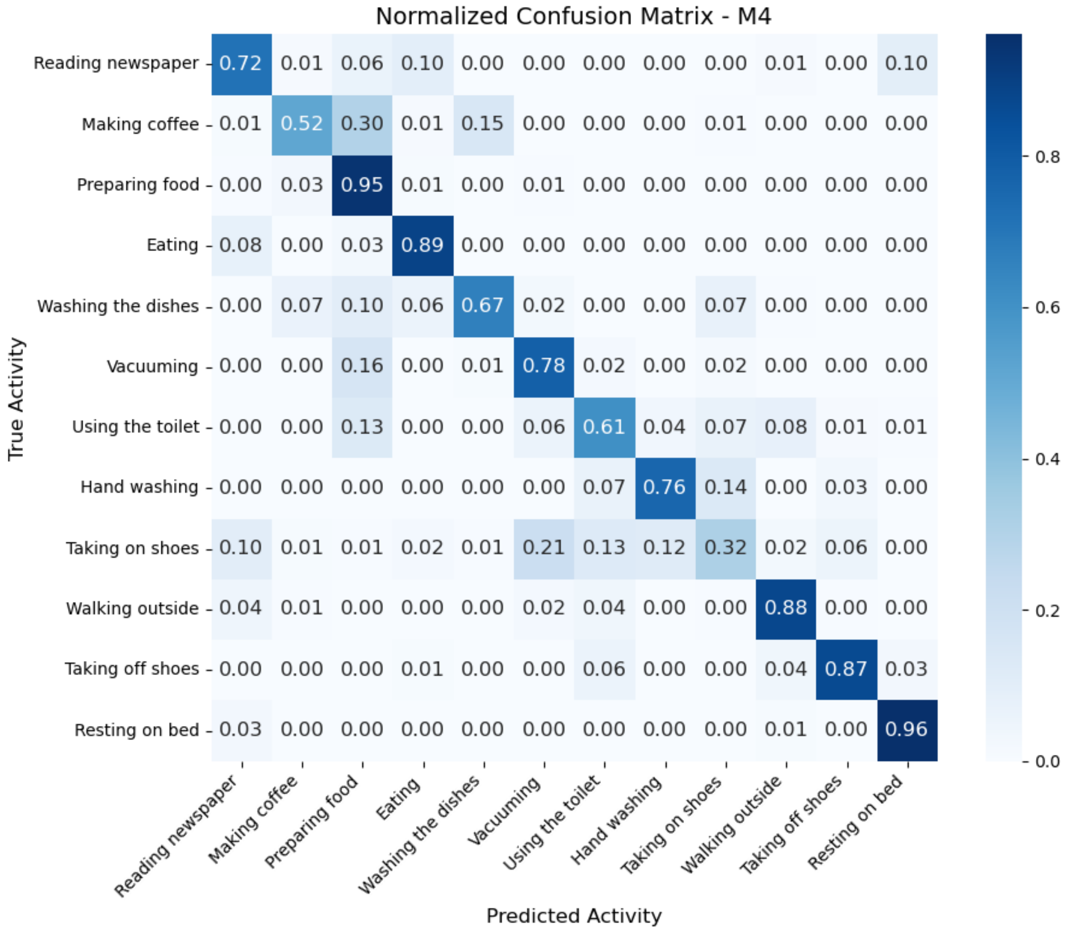

True \ Predicted	Reading newspaper	Making coffee	Preparing food	Eating	Washing the dishes	Vacuuming	Using the toilet	Hand washing	Taking on shoes	Walking outside	Taking off shoes	Resting on bed
Reading newspaper	0.72	0.01	0.06	0.10	0.00	0.00	0.00	0.00	0.00	0.01	0.00	0.10
Making coffee	0.01	0.52	0.30	0.01	0.15	0.00	0.00	0.00	0.01	0.00	0.00	0.00
Preparing food	0.00	0.03	0.95	0.01	0.00	0.01	0.00	0.00	0.00	0.00	0.00	0.00
Eating	0.08	0.00	0.03	0.89	0.00	0.00	0.00	0.00	0.00	0.00	0.00	0.00
Washing the dishes	0.00	0.07	0.10	0.06	0.67	0.02	0.00	0.00	0.07	0.00	0.00	0.00
Vacuuming	0.00	0.00	0.16	0.00	0.01	0.78	0.02	0.00	0.02	0.00	0.00	0.00
Using the toilet	0.00	0.00	0.13	0.00	0.00	0.06	0.61	0.04	0.07	0.08	0.01	0.01
Hand washing	0.00	0.00	0.00	0.00	0.00	0.00	0.07	0.76	0.14	0.00	0.03	0.00
Taking on shoes	0.10	0.01	0.01	0.02	0.01	0.21	0.13	0.12	0.32	0.02	0.06	0.00
Walking outside	0.04	0.01	0.00	0.00	0.00	0.02	0.04	0.00	0.00	0.88	0.00	0.00
Taking off shoes	0.00	0.00	0.00	0.01	0.00	0.00	0.06	0.00	0.00	0.04	0.87	0.03
Resting on bed	0.03	0.00	0.00	0.00	0.00	0.00	0.00	0.00	0.00	0.01	0.00	0.96

Supplementary Figure 5. Normalised confusion matrix for Model 4 (M4) across 12 everyday activities.
