# Supplementary material for: Recognition of everyday activities using experiment data from wearable sensors: a deep learning-based framework
Source: Sci Rep. 2026 Jul 24;16:23218. doi: 10.1038/s41598-026-63774-8 (PMC13400646; doi:10.1038/s41598-026-63774-8)
Supplement: Supplementary file 3 — Supplementary Material 3 [file 41598_2026_63774_MOESM3_ESM.pdf]

Supplementary Table 2. Comparison of computational performance and model complexity metrics for LSTM, Logistic Regression (LR), Random Forest (RF), and Support Vector Machine (SVM), including training time, inference time, model size, and structural complexity indicators.

Algorithm	*Train time (sec)*	*Predict time (sec)*	*Predict time per sample (ms)*	*Trainable params*	*Model size (mb)*	*N trees*	*Total nodes*	*N support vectors*
LSTM	487,0091474	2,369911671	1,795388	308014	1,176506042			
LR	9,753605843	0,00060463	0,000398	2534	0,020102501			
RF	18,02735353	0,015272617	0,010048		46,72304249	100	278120	
SVM	27,19093156	3,024322987	1,989686		22,20057678			15036

Table 2 compares the computational efficiency and complexity of the evaluated machine learning models. Although the LSTM model required substantially longer training time (487.01 s) than conventional machine learning models, its selection is justified by its ability to learn complex temporal and non-linear relationships inherent in the sequential nature of our data. Unlike Logistic Regression, Random Forest, and SVM, which primarily rely on handcrafted features and static input representations, LSTM networks incorporate memory cells and gating mechanisms that enable them to capture long-term dependencies and contextual information. The resulting model size remained relatively modest (1.18 MB) despite containing 308,014 trainable parameters, indicating efficient parameter storage.
